# Extracellular Water to Total Body Water Ratio in Septic Shock Patients Receiving Protocol-Driven Resuscitation Bundle Therapy

**DOI:** 10.3390/jcm10132917

**Published:** 2021-06-29

**Authors:** Bora Chae, Yo Sep Shin, Seok-In Hong, Sang Min Kim, Youn-Jung Kim, Seung Mok Ryoo, Won Young Kim

**Affiliations:** Department of Emergency Medicine, University of Ulsan College of Medicine, Asan Medical Center, 88, Olympic-ro 43-gil, Songpa-gu, Seoul 05505, Korea; brchae@gmail.com (B.C.); irrusters@gmail.com (Y.S.S.); finefigs@gmail.com (S.-I.H.); swdarkhorse@gmail.com (S.M.K.); yjkim.em@gmail.com (Y.-J.K.); chrisyoo@gmail.com (S.M.R.)

**Keywords:** septic shock, body fluid composition, bio-electrical impedance analysis, ECW/TBW, mortality

## Abstract

(1) Bio-electrical impedance analysis (BIA) is a rapid, simple, and noninvasive tool for evaluating the metabolic status and for assessing volume status in critically ill patients. Little is known, however, the prognostic value of body composition analysis in septic shock patients. This study assessed the association between parameters by body composition analysis and mortality in patients with septic shock in the emergency department (ED). (2) Data were prospectively collected on adult patients with septic shock who underwent protocol-driven resuscitation bundle therapy between December 2019 and January 2021. The primary outcome was 30-day mortality. (3) The study included 261 patients, the average ratio of extracellular water (ECW) to total body water (TBW) was significantly higher in non-survivors than in survivors (0.414 vs. 0.401, *p* < 0.001). Multivariate analysis showed that ECW/TBW ≥ 0.41 (odds ratio (OR), 4.62; 95% confidence interval (CI), 2.31–9.26, *p* < 0.001), altered mental status (OR, 2.88; 95% CI, 1.28–6.46, *p* = 0.010), and lactate level (OR, 1.24; 95% CI, 1.12–1.37, *p* < 0.001) were significantly associated with 30-day mortality in patients with septic shock. (4) ECW/TBW ≥ 0.41 may be associated with 30-day mortality in patients with septic shock receiving protocol-driven resuscitation bundle therapy in the ED.

## 1. Introduction

Septic shock, which is caused by dysregulation of a host response to infection, is a life-threatening medical condition with high morbidity and mortality rates. In addition to early recognition, guidelines for the management of septic shock recommend the application of protocol-driven resuscitation bundle therapy, which includes fluid resuscitation, blood culturing, and the administration of broad-spectrum antibiotics [1].

Despite these supportive therapies, mortality rates remain high, suggesting the need to identify modifiable factors or targets for adjuvant therapies in patients with septic shock [2,3]. Bio-electrical impedance analysis (BIA) of body composition is a non-invasive method of differentiating among fats, proteins, and minerals. The BIA machines used these days use only impedance to determine body composition, so empirical estimations such as age or gender do not affect to results. In addition, multifrequency BIA is a rapid, simple, and reproducible method for evaluating the metabolic status and for assessing volume status with extracellular and intracellular water in critically ill patients [4,5,6]. Thus, body composition analysis using BIA may provide useful information in patients undergoing dialysis, in burn patients, and in patients with malnutrition, trauma, and other critical illnesses [7,8,9,10,11]. Little is known, however, about the prognostic value of body composition analysis in septic shock patients during early resuscitation period. This study hypothesized that body composition analysis might provide information regarding abnormal fluid and nutritional status associated with mortality, particularly during the early resuscitation period.

The aim of this study was to determine the association between parameters identified by body composition analysis and mortality in patients with septic shock who underwent protocol-driven resuscitation bundle therapy in the emergency department (ED).

## 2. Materials and Methods

### 2.1. Study Design and Population

This prospective observational study included adult patients, aged ≥18 years, with septic shock who were admitted to the ED of a tertiary referral center in Seoul, Korea, between December 2019 and December 2020 and underwent protocol-driven resuscitation care bundle therapy and measurements of body composition by BIA. The protocol of bundle therapy for septic shock patients in our institution was described in Supplementary S1. Septic shock was based on sepsis-3 criteria, defined as refractory hypotension, hyperlactatemia (≥2 mmol/L), and suspected or confirmed infection [12]. Refractory hypotension was defined as persistent hypotension with systolic blood pressure <90 mmHg or mean arterial pressure <70 mmHg or the need for vasopressors despite adequate intravenous fluid resuscitation (20–30 mL/kg or ≥1 L of crystalloid solution administration) [13,14]. Active cancer was defined as a histologically confirmed solid or hematologic malignancy that had been diagnosed or treated within the previous 6 months, or as a recurrent, regionally advanced, or metastatic cancer treated within the previous 6months [15,16]. If a patient visited the ED multiple times during the study period, only the first visit was included. The study design was approved by the Institutional Review Board of Asan Medical Center and all patients gave written informed consent before the participant enters the research (Supplementary S2). The primary outcome of this study was 30-day mortality, and all patients were followed up for more than 30 days.

### 2.2. Data Collection

Patients’ electronic medical records were reviewed, and their demographic, clinical, and laboratory test results on ED admission were recorded. Details of each patient’s medical history, including hypertension, diabetes mellitus, chronic renal disease, chronic liver disease, heart failure, cardiovascular disease, and active cancer, were also recorded. Laboratory test results during ED stay included leukocyte and platelet counts; and hemoglobin (Hb), creatinine, albumin, total bilirubin, C-reactive protein (CRP), and lactate levels. Mental status was assessed using the alert/responsive to voice/responsive to pain/unresponsive scale, and patients who were not alert were considered to have altered mental status [17]. Sequential Organ Failure Assessment (SOFA) scores were calculated based on physiological and laboratory data collected in the ED [18].

BIA was performed using a portable multifrequency bio-impedance device (InBody S10, InBody Co. Ltd., Seoul, Korea) to differentiate tissues such as protein, fat, muscle, mineral, and body water content based on their electrical impedances. This analyzer measured each of the body’s five parts (left arm, right arm, trunk, left leg, and right leg) as segmental resistance at six frequencies (1, 5, 50, 250, 500 kHz, and 1 MHz). The difference in frequencies of current is related to their abilities to penetrate cell membranes. Due to this, multi-frequency current able to differentiate the proportion of the conductive component, the water. Therefore, developed multi-frequencies BIA device accurately measure the distribution of body composition com-paring the single frequency method (Supplementary S3)

Body composition analyses by the BIA device were carried out in the following order. Patients recognized as septic shock are required to maintain the supine posture at least 10 min before applying the device. Place 4 units of touch-type hand electrodes on the thumb and middle fingers of both hands and 2 units of touch-type foot electrodes between ankle bone and heel. Make the examinee keep both arms abducted naturally to a 15-degree angle away from the trunk with a supine position. Press the ‘Enter’ button to start the test, then values in body composition including the ECW/TBW ratio are calculated and saved automatically. Utilizing the BIA device is easy and simple, so the examiner does not require a high degree of technical skill to operate. The average time for preparation of the device, adjustment, and measurement is about 10 min (Supplementary S3) [19,20,21]. Body composition analyses were performed during resuscitation for septic shock. All BIA tests were carried out by interns who were familiar with how to use the device according to the manufacturer’s protocol.

### 2.3. Statistical Analysis

Categorical variables were reported as numbers and percentages and compared using the chi-square test or Fisher’s exact test, as appropriate. Continuous variables were reported as means ± standard deviations due to their normal distribution and compared using Student’s t-test or the Wilcoxon rank-sum test. Factors associated with 30-day mortality were assessed by univariate and multivariate logistic regression analyses. Variables with *p* < 0.1 on univariate analyses were included in the multivariable analyses. The logistic model of goodness of fit was evaluated using the Hosmer–Lemeshow test. The results of multivariate logistic regression analysis were reported as odds ratios (OR) and 95% confidence intervals (CI). The optimal cutoff value of variables for predicting 30-day mortality was estimated by receiver operating characteristic (ROC) curves. For all tests, *p* values were two tailed, and those <0.05 were considered statistically significant. All analyses were performed using IBM SPSS Statics for Windows, version 21.0 (IBM Corp., Armonk, NY, USA).

## 3. Results

### 3.1. Baseline Characteristics of Total Patients

**Figure 1:**
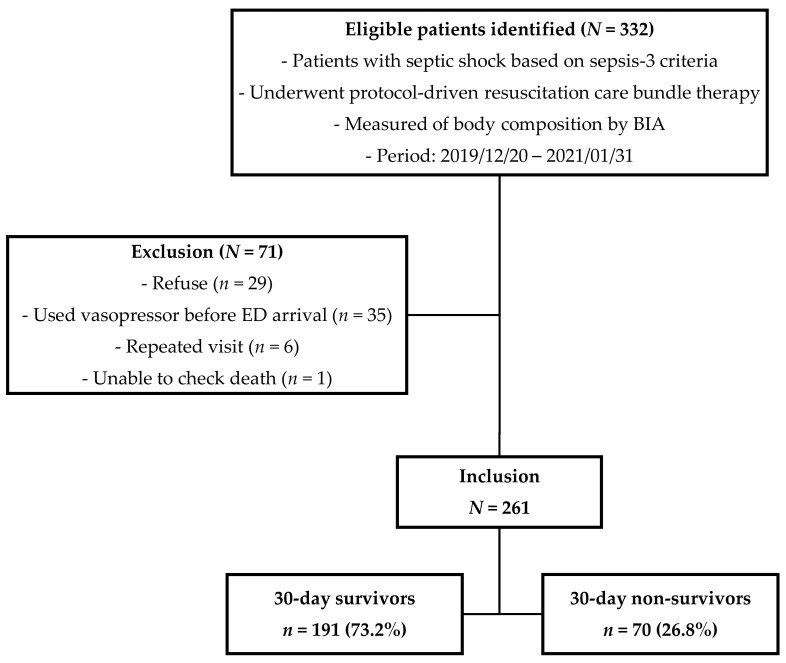
Patient flow diagram. BIA, Bio-electrical impedance analysis; ED, emergency department. Patients with septic shock based on sepsis-3 criteria who underwent protocol-driven resuscitation care bundle therapy and measurements of body composition by Bio-electrical impedance analysis. From December 2019 to January 2021, 261 patients were enrolled and the overall 30-day mortality rate was 26.8%.

During the study period, from December 2019 to January 2021, 261 patients with septic shock and who underwent BIA in the ED were enrolled, as shown in Figure 1. The overall 30-day mortality rate was 26.8%.

The demographic and clinical characteristics of 30-day survivors and non-survivors are compared in Table 1. Mean age was 66.7 years and 59.0% were male. Age, sex, and medical history did not differ significantly in the two groups, but active cancer was significantly lower in survivors than in non-survivors (61.3% vs. 77.1%, *p* = 0.017). The average Charlson comorbidity index in the total population was 5.02, it was significantly higher in non-survivors than in survivors (6.03 vs. 4.65, *p* = 0.003). Heart rate (HR) was significantly higher in non-survivors than in survivors (114.2 vs. 107.2 bpm, *p* = 0.025). Altered mental status was more common in non-survivors than in survivors and the difference was statistically significant (28.6% vs. 13.6%, *p* = 0.005). Evaluation of laboratory data showed that Hb levels were significantly lower (9.91 vs. 10.78 g/dL, *p* = 0.011), whereas while lactate levels (5.70 vs. 3.39 mmol/L, *p* < 0.001), and initial SOFA scores (6.33 vs. 4.57, *p* < 0.001) were significantly higher, in non-survivors than in survivors. No patient was receiving the vasopressors with the initial lactate level was above 4 mmol/L at the time of ED presentation. Total 84% of patients were receiving the vasopressor with follow-up second lactate level was above 4 mmol/L at the end of resuscitation. The maximum dose of norepinephrine during the admission was significantly higher in the non-survivors than survivors (0.21 vs. 0.15 mcg/kg/min, *p* = 0.001). Ventilator use (28.6% vs. 13.6%, *p* = 0.005) and the application of continuous renal replacement therapy (CRRT; 20.0% vs. 6.3%, *p* = 0.001) were significantly more frequent in non-survivors than in survivors. For additional information on the resuscitation course, we provided the compliance of protocol and the timing of each factor in bundle therapy in Supplementary S1.

### 3.2. Body Composition Analysis of Total Population

Table 2 summarizes the results of body composition analysis in 30-day survivors and non-survivors. The mean time from ED admission to the measurement of body composition was 5.2 h. The total volume of administrated fluid before measurement of body composition was 29.8 cc/kg. The type of given fluid was normal saline and a balanced solution. Blood product such as packed red blood cells, fresh frozen plasma, platelet concentrates, and cryoprecipitates were administrated with the crystalloid for resuscitation if needed. The average ratio of ECW/TBW ratio was significantly higher in non-survivors than in survivors (0.414 ± 0.029 vs. 0.401 ± 0.022, *p* < 0.001). The prevalence of patients with ECW/TBW ≥ 0.41 was significantly higher in the non-survivor than in the survivor group (64.3% vs. 28.3%, *p* < 0.001). No other body composition parameter along with the phase angle (PA) values differed significantly except PA at 50 kHz left leg between survivors and non-survivors.

### 3.3. Characteristics of Septic Shock Patients with Higher ECW/TBW

Table 3 compares the baseline characteristics of patients with ECW/TBW ≥ 0.41 and <0.41. The percentage of male (47.5% vs. 66.0%, *p* = 0.003) was lower in the group with ECW/TBW ≥ 0.41 than in that with ECW/TBW <0.41. Charlson co-morbidity index was significantly higher in the group with ECW/TBW ≥ 0.41 than in that with ECW/TBW <0.41 (5.60 vs. 4.67. *p* = 0.031). Body temperature (37.09 vs. 38.47 °C, *p* = 0.034), Hb levels (9.85 vs. 10.97 g/dL, *p* < 0.001), and albumin (1.96 vs. 2.06 g/dL, *p* < 0.001) were significantly lower in the ECW/TBW ≥ 0.41 group than in the ECW/TBW <0.41 group. The 30-day mortality (45.5% vs. 15.4%, *p* < 0.001) and initial SOFA scores (5.67 vs. 4.66, *p* = 0.004) were significantly higher in patients with ECW/TBW ≥ 0.41 than in patients with ECW/TBW < 0.41.

### 3.4. Risk Factors for 30-Day Mortality in Patients with Septic Shock

Univariate and multivariate logistic regression analyses were performed to identify the risk factors for 30-day mortality in patients with septic shock (Table 4). Age, Sex, and variables with *p* < 0.1 in univariate analysis, such as ECW/TBW ≥ 0.41, active cancer, HR, altered mental status, Hb, albumin, and lactate levels, were included in the multivariable analyses. Multivariate analysis found that ECW/TBW ≥ 0.41 (OR, 4.62; 95% confidence interval [CI], 2.31–9.26, *p* < 0.001), altered mental status (OR, 2.88; 95% CI, 1.28–6.46, *p* = 0.010), and lactate level (OR, 1.24; 95% CI, 1.12–1.37, *p* < 0.001) were independently associated with 30-day mortality in patients with septic shock.

## 4. Discussion

The present study found that the ECW/TBW was significantly higher in 30-day non-survivors than in 30-day survivors of septic shock (0.414 vs. 0.401, *p* = 0.001). Septic shock patients with higher ECW/TBW were more likely to have lower Hb concentrations and albumin than patients with lower ECW/TBW. To our knowledge, this is the first study to investigate the association between ECW/TBW and mortality of patients with septic shock.

BIA is an objective method that measures and analyzes body composition by sending a weak electrical current through the body. It is also a reproducible method that can be performed at the bedside. Its efficacy and accuracy in predicting body water composition are comparable to those of more classic methods [22,23]. Thus, multi-frequency BIA can be used to assess the volume and nutritional status of patients with various diseases [24,25,26]. Furthermore, several studies have reported significant relationships between BIA-determined imbalances in body fluid and clinical outcomes in patients with, for example, chronic renal failure, chronic liver disease, and chronic obstructive pulmonary disease [26,27,28]. Extracellular fluid retention may play an important role in the progression and deterioration of diseases, indicating that pathophysiologic alterations in body fluid composition are associated with poor clinical outcomes [29,30,31].

ECW/TBW as determined by BIA is frequently used to assess abnormal fluid status, [25,32] and is a sensitive indicator of hydration changes [33]. Higher ECW/TBW ratios have been reported to predict clinical outcomes in patients with heart failure, liver diseases, renal disorders, and malignancies [24,25,26,34]. Alterations in body fluid distribution without effective volume expansion result in excess fluid retention in the extracellular space, which can cause poor outcomes in critically ill patients [35,36]. The present study found that ECW/TBW was the only statistically significant body composition variable associated with mortality in patients with septic shock.

Inflammatory processes during septic shock induce endothelial damage, increasing vascular permeability and shifting fluid from the intracellular to the extracellular space [37,38,39]. These alterations in body water distribution, such as ECW expansion, exacerbate the deterioration of cell membrane function [40]. In responding to cardiac dysfunction during septic shock, fluid retention volume may be increased by fluid resuscitation [41]. This accumulation of fluid in a third space leads to a vicious cycle, in which the patient’s condition deteriorates, further contributing to fluid retention during septic shock.

The normal range of ECW/TBW is between 0.360–0.390, with ratios ≥ 0.400 indicating an overhydrated state [42,43]. The present study found that the ECW/TBW was significantly higher in 30-day non-survivors than in 30-day survivors of septic shock (0.414 vs. 0.401, *p* = 0.001). Septic shock patients with higher ECW/TBW were more likely to have lower Hb concentrations and albumin than patients with lower ECW/TBW. A previous study reported the average of ECW/TBW in patients with bacteremia was 0.510, suggesting that ECW/TBW can be higher in various conditions associated with reduced lean muscle mass, not only in septic shock [44,45,46,47]. The average ECW/TBW was found to be 0.42 in non-survivors admitted to the intensive care unit, patients who tended to be malnourished [35]. Average ECW/TBW ratios were found to be 0.412 in patients with acute heart failure [26] and 0.40 in chronic dialysis patients [48]. Although these averages were higher than the normal reference value, they were lower in patients with chronic diseases than in those who were critically ill.

Fluid resuscitation and administration of multiple drugs to septic shock patients can result in faster and more dynamic hemodynamic changes. BIA is not only a prognostic tool but may be an effective and objective method of assessing real-time hemodynamic parameters at the bedside. Most importantly, BIA can repeatedly provide useful information about body fluid distribution or hydration status. Further studies are required to evaluate co-morbidities that can affect body fluid composition, enabling more accurate prediction of patient prognosis and determining whether fluid resuscitation is warranted in patients with septic shock.

The major limitation of this study was that it involved patients at a single center. In addition, the methodological limitation of the BIA device performing to patients with septic shock could not control completely. Patients with discomfort, aging, and alteration of mental status made an unintentional movement or could not maintain sufficient proper posture for analysis. Additionally, there were various considerable factors that can affect the results of BIA. The flatness of floor, the distance of power supply from equipment, the timing of fluid loading or BIA measurement, the presence of other electrical devices, and excessively high or low skin temperature or humidity of skin, wet bedding due to urination or sweating, and prior oral intake or medication such as diuretics could be sources of data disruption. Furthermore, the results of BIA may have been confounded by co-morbidities, such as chronic liver disease, chronic renal disease, and malignancies.

## 5. Conclusions

ECW/TBW ≥ 0.41 was associated with 30-day mortality in patients with septic shock receiving protocol-driven resuscitation bundle therapy in the ED. Further studies considering factors that affect the results of BIA are needed to determine appropriate fluid resuscitation strategies for septic shock.

## Figures and Tables

**Table 1:** Comparison of the clinical characteristics between the 30-day non-survivor group and the 30-day survivor group.

	Total (*n* = 261)	30-Day Survivors (*n* = 191)	30-Day Non-Survivors (*n* = 70)	*p* Value
Age (years)	66.66 ± 11.49	66.55 ± 11.25	66.94 ± 12.18	0.807
Sex—Male	154 (59.0)	111 (58.1)	43 (61.4)	0.630
BMI (kg/m^2^)	21.79 ± 3.75	21.82 ± 3.64	21.68 ± 4.08	0.785
**Comorbidities (%)**				
Hypertension	90 (34.5)	68 (35.6)	22 (31.4)	0.530
Diabetes mellitus	75 (28.7)	61 (31.9)	14 (20.0)	0.059
Chronic renal disease	24 (9.2)	18 (9.4)	6 (8.6)	0.833
Cardiovascular disease	28 (10.7)	19 (9.9)	9 (12.9)	0.501
Heart failure	13 (5.0)	8 (4.2)	5 (7.1)	0.348
Chronic liver disease	36 (13.8)	26 (13.6)	10 (14.3)	0.889
Malignancy	171 (65.5)	117 (61.3)	54 (77.1)	0.017
Charlson co-morbidity index	5.02 ± 3.36	4.65 ± 3.30	6.03 ± 3.34	0.003
**Vital sign**				
SBP (mmHg)	88.57 ± 22.12	89.60 ± 23.07	85.74 ± 19.13	0.212
DBP (mmHg)	57.22 ± 15.27	57.92 ± 14.98	55.30 ± 16.00	0.220
Heart rate (bpm)	109.2 ± 23.75	107.2 ± 23.14	114.2 ± 24.68	0.025
Body temperature (°C)	37.95 ± 5.13	37.74 ± 1.40	38.53 ± 9.66	0.494
SpO_2_ (%)	94.49 ± 5.87	94.78 ± 4.67	93.71 ± 8.29	0.309
Altered mental status	46 (17.6)	26 (13.6)	20 (28.6)	0.005
**Laboratory data**				
WBC (×10^3^/μL)	11.31 ± 9.80	10.97 ± 8.93	12.25 ± 11.88	0.415
Hemoglobin (g/dL)	10.55 ± 2.45	10.78 ± 2.43	9.91 ± 2.42	0.011
Platelet (×10^3^/μL)	155.4 ± 112.9	158.4 ± 103.53	147.2 ± 135.82	0.532
Creatinine (mg/dL)	2.02 ± 1.68	1.94 ± 1.68	2.26 ± 1.65	0.162
Albumin (g/dL)	2.59 ± 1.03	2.65 ± 0.60	2.42 ± 1.72	0.120
Total bilirubin (mg/dL)	2.54 ± 4.33	2.17 ± 3.32	3.57 ± 6.23	0.078
CRP (mg/dL)	14.95 ± 10.29	14.64 ± 10.34	15.79 ± 10.20	0.428
Lactate (mmol/L)	4.16 ± 3.11	3.53 ± 2.72	5.87 ± 3.45	<0.001
Initial SOFA	5.04 ± 2.77	4.57 ± 2.50	6.33 ± 3.04	<0.001
SOFA day 1	9.29 ± 3.78	8.47 ± 3.27	11.53 ± 4.18	<0.001
**At the time of ED presentation (%) ^†^**				
Vasopressor used	4 (1.5)	4 (2.1)	0 (0.0)	0.222
Lactate > 4 mmol/L	98 (37.5)	50 (26.2)	48 (68.6)	<0.001
Vasopressor used and Lactate > 4 mmol/L	0 (0.0)	(0.0)	(0.0)	NA
**At the end of resuscitation (%) ^††^**				
Vasopressor used	261 (100.0)	191 (100.0)	70 (100.)	NA
Lactate > 4 mmol/L	84 (32.2)	47 (24.6)	37 (52.9)	<0.001
Vasopressor used and Lactate > 4 mmol/L	84 (32.2)	47 (24.6)	37 (52.9)	<0.001
NE, maximum (mcg/kg/min) ^†††^	0.17 ± 0.12	0.15 ± 0.12	0.21 ± 0.14	0.001
Ventilator	46 (17.6)	26 (13.6)	20 (28.6)	0.005
CRRT	26 (10.0)	12 (6.3)	14 (20.0)	0.001

Values are expressed as the mean ± standard deviation, the median (interquartile range) or number (%). BMI, body mass index; SBP, systolic blood pressure; DBP, diastolic blood pressure; SpO_2_, saturation of percutaneous oxygen; WBC, white blood cell; CRP, c-reactive protein; SOFA, sequential organ failure assessment; ED, emergency department; NA, not applicable;NE, norepinephrine; CRRT, continuous renal replacementtherapy; ^†^ At the time of ED presentation, the time of patients arrive at ED. ^††^ At the end of resuscitation, the time after the second lactate level was obtained. ^†††^ NE, maximum, maximum dose of norepinephrine administered during admission from the recognized as a septic shock at the ED to discharge or death.

**Table 2:** Comparison of the body composition between the 30-day non-survivor group and the 30-day survivor group.

	Total (*n* = 261)	30-Day Survivors (*n* = 191)	30-Day Non-Survivors (*n* = 70)	*p* Value
Time to body composition (hour) ^†^	5.24 ± 3.70	5.30 ± 3.76	5.08 ± 3.56	0.676
Administrated fluid (cc/kg) ^††^	29.8 ± 11.6	30.4 ± 11.4	28.1 ± 11.9	0.139
**Fluid type (%)**				
Normal saline	137 (52.5)	98 (51.3)	39 (55.7)	0.528
Balanced solution	124 (47.5)	93 (48.7)	31 (44.3)	
Additional blood product	9 (3.4)	2 (1.0)	7 (10.0)	<0.001
ICW (L)	19.82 ± 4.36	19.98 ± 4.44	19.40 ± 4.14	0.342
ECW (L)	13.43 ± 2.90	13.35 ± 2.95	13.65 ± 2.75	0.454
TBW (L)	33.25 ± 7.04	33.32 ± 7.23	33.05 ± 6.53	0.779
Protein (Kg)	8.57 ± 1.88	8.64 ± 1.92	8.38 ± 1.79	0.325
Mineral (Kg)	3.17 ± 0.72	3.18 ± 0.66	3.17 ± 0.86	0.963
Fat (Kg)	12.40 ± 8.30	12.29 ± 7.94	12.67 ± 9.28	0.746
Soft lean mass (Kg)	42.35 ± 9.01	42.50 ± 9.24	41.97 ± 8.37	0.674
Fat free mass (Kg)	44.99 ± 9.48	45.14 ± 9.71	44.60 ± 8.90	0.685
Skeletal muscle mass (Kg)	23.85 ± 5.68	24.05 ± 5.78	23.30 ± 5.40	0.346
Percent body fat (%)	20.88 ± 11.83	20.75 ± 11.59	21.24 ± 12.87	0.769
Waist-hip ratio	0.75 ± 0.12	0.76 ± 0.10	0.76 ± 0.15	0.107
ECW/TBW	0.404 ± 0.025	0.401 ± 0.022	0.414 ± 0.029	<0.001
ECW/TBW ≥ 0.41	99 (37.9)	54 (28.3)	45 (64.3)	<0.001
Body cell mass	28.39 ± 6.24	28.61 ± 6.35	27.78 ± 5.93	0.343
Bone mineral content (Kg)	2.64 ± 0.63	2.64 ± 0.57	2.63 ± 0.78	0.973
5 kHz RA phase angle (°)	1.74 ± 3.21	1.84 ± 3.72	1.49 ± 0.80	0.449
5 kHz LA phase angle (°)	1.46 ± 0.87	1.52 ± 0.97	1.33 ± 0.46	0.118
5 kHz TR phase angle (°)	2.50 ± 2.28	2.50 ± 2.27	2.47 ± 2.29	0.927
5 kHz RL phase angle (°)	2.07 ± 1.52	2.10 ± 1.24	1.97 ± 2.10	0.622
5 kHz LL phase angle (°)	2.40 ± 2.62	2.49 ± 2.52	2.16 ± 2.89	0.367
50 kHz RA phase angle (°)	4.13 ± 2.07	4.18 ± 2.02	4.00 ± 2.19	0.532
50 kHz LA phase angle (°)	4.01 ± 3.23	4.16 ± 3.70	3.62 ± 1.14	0.235
50 kHz TR phase angle (°)	3.04 ± 2.85	3.11 ± 2.43	2.85 ± 3.79	0.600
50 kHz RL phase angle (°)	4.39 ± 2.80	4.58 ± 2.55	3.88 ± 3.35	0.070
50 kHz LL phase angle (°)	4.36 ± 2.99	4.60 ± 2.69	3.69 ± 3.64	0.029
250 kHz RA phase angle (°)	6.38 ± 2.72	6.13 ± 1.66	7.06 ± 4.44	0.092
250 kHz LA phase angle (°)	5.98 ± 2.74	5.90 ± 2.40	6.21 ± 3.52	0.420
250 kHz TR phase angle (°)	2.18 ± 4.21	2.15 ± 3.79	2.25 ± 5.22	0.870
250 kHz RL phase angle (°)	4.35 ± 3.24	4.53 ± 3.32	3.85 ± 3.00	0.139
250 kHz LL phase angle (°)	4.26 ± 3.39	4.44 ± 3.41	3.76 ± 3.28	0.149

Values are expressed as the mean ± standard deviation, the median (interquartile range) or number (%). ICW, intracellular water; ECW, extracellular water; TBW, total body water; RA, right arm; LA, left arm; TR, trunk; RL, right leg; LL, left leg; kHz, kilohertz. ^†^ Mean time to body composition, time duration from the emergency department admission to the measurement of body composition. ^††^ Administrated fluid, initial dripped fluid volume for resuscitation as a protocol-driven bundle therapy of septic shock.

**Table 3:** Comparison of the clinical characteristics based on the ECW/TBW.

	Total (*n* = 261)	ECW/TBW < 0.41 (*n* = 162)	ECW/TBW ≥ 0.41 (*n* = 99)	*p* Value
Age (years)	66.66 ± 11.49	65.44 ± 11.66	67.53 ± 11.32	0.148
Sex—Male	154 (59.0)	107 (66.0)	47 (47.5)	0.003
**Comorbidities**				
Hypertension	90 (34.5)	56 (34.6)	34 (34.3)	0.970
Diabetes mellitus	75 (28.7)	47 (29.0)	28 (28.3)	0.899
Chronic renal disease	24 (9.2)	13 (8.0)	11 (11.1)	0.402
Cardiovascular disease	28 (10.7)	19 (11.7)	9 (9.1)	0.504
Heart failure	13 (5.0)	7 (4.3)	6 (6.1)	0.531
Chronic liver disease	36 (13.8)	20 (12)	16 (16.2)	0.386
Malignancy	171 (65.5)	102 (63.0)	69 (69.7)	0.267
Charlson co-morbidity index	5.02 ± 3.36	4.67 ± 3.42	5.60 ± 3.19	0.031
**Vital sign**				
SBP (mmHg)	88.57 ± 22.12	90.41 ± 23.49	85.56 ± 19.41	0.086
DBP (mmHg)	57.22 ± 15.27	58.59 ± 16.60	54.97 ± 12.57	0.063
Heart rate (bpm)	109.17 ± 23.75	107.78 ± 24.49	111.44 ± 22.41	0.228
Body temperature (°C)	37.95 ± 5.13	38.47 ± 6.37	37.09 ± 1.38	0.034
SpO_2_ (%)	94.49 ± 5.87	94.74 ± 6.18	94.09 ± 5.31	0.388
Altered mental status	46 (17.6)	25 (15.4)	21 (21.2)	0.234
**Laboratory data**				
WBC (×10^3^/μL)	11.31 ± 9.80	10.24 ± 8.75	13.06 ± 11.14	0.033
Hemoglobin (g/dL)	10.55 ± 2.45	10.97 ± 2.45	9.85 ± 2.30	<0.001
Platelet (×10^3^/μL)	155.36 ± 112.9	158.57 ± 109.8	150.11 ± 118.2	0.558
Creatinine (mg/dL)	2.02 ± 1.68	2.06 ± 1.81	1.96 ± 1.43	0.618
Albumin (g/dL)	2.59 ± 1.03	2.80 ± 1.20	2.24 ± 0.50	<0.001
Total bilirubin (mg/dL)	2.54 ± 4.33	2.45 ± 4.39	2.70 ± 4.24	0.642
CRP (mg/dL)	14.95 ± 10.29	15.47 ± 10.88	14.11 ± 9.38	0.302
Lactate (mmol/L)	5.23 ± 4.64	3.68 ± 2.91	4.55 ± 3.50	0.039
30-day mortality	70 (26.8)	25 (15.4)	45 (45.5)	<0.001
Initial SOFA	5.04 ± 2.77	4.66 ± 2.60	5.67 ± 2.92	0.004
SOFA day 1	9.29 ± 3.78	8.80 ± 3.89	10.09 ± 3.47	0.007
Ventilator	46 (17.6)	27 (16.7)	19 (19.2)	0.603
CRRT	26 (10.0)	15 (9.3)	11 (11.1)	0.628

Values are expressed as the mean ± standard deviation, the median (interquartile range) or number (%). SBP, systolic blood pressure; DBP, diastolic blood pressure; SpO_2_, saturation of percutaneous oxygen; WBC, white blood cell; CRP, c-reactive protein; SOFA, sequential organ failure assessment; CRRT, continuous renal replacement therapy.

**Table 4:** Univariate and multivariate logistic regression analysis for the association of mortality in septic shock.

	Univariate		Multivariate	
	OR (95% CI)	*p* Value	OR (95% CI)	*p* Value
Age	1.003 (0.979–1.027)	0.806		
Male	1.148 (0.655–2.011)	0.630		
ECW/TBW ≥ 0.41	2.897 (1.565–5.362)	0.001	4.621 (2.305–9.263)	<0.001
Malignancy	2.135 (1.138–4.005)	0.018	1.752 (0.790–3.884)	0.168
Heart rate	1.014 (1.002–1.026)	0.026	1.012 (0.998–1.026)	0.094
Altered mental status	2.538 (1.308–4.928)	0.006	2.881 (1.284–6.461)	0.010
Hemoglobin	0.860 (0.765–0.968)	0.012	0.895 (0.770–1.041)	0.150
Albumin	0.646 (0.405–1.033)	0.068	1.120 (0.851–1.474)	0.419
Lactate	1.243 (1.136–1.360)	<0.001	1.241 (1.122–1.373)	<0.001

OR, odds ratio; CI, confidence interval; ECW, extracellular water; TBW, total body water; SBP, systolic blood pressure; DBP, diastolic blood pressure. Hosmer and Lemeshow Goodness of Fit test: χ2 = 11.164, df = 8, *p*-value 0.193.

## Data Availability

No new data were created or analyzed in this study. Data sharing is not applicable to this article.

## Supplementary Materials

The following are available online at https://www.mdpi.com/article/10.3390/jcm10132917/s1, Title: Supplementary S1. The protocol of bundle therapy for septic shock patients and compliance of our institution.; Supplementary S2. Explanation and informed consent about study registration of patients with septic shock at the emergency department.; Supplementary S3. BIA device and protocol.

## Abbreviations

BIA	Bio-electrical impedance analysis
ED	Emergency department
CBC	Complete blood cell counts
CRP	C-reactive protein
SOFA	Sequential Organ Failure Assessment
kHz	Kilohertz
ECW	Extracellular water
TBW	Total body water
OR	Odds ratios
CI	Confidence intervals
ROC	Receiver operating characteristic
Hb	Hemoglobin
BMI	Body mass index
SBP	Systolic blood pressure
DBP	Diastolic blood pressure
SpO_2_	Saturation of percutaneous oxygen
WBC	White blood cell
CRRT	Continuous renal replacement therapy
PA	Phase angle
ICW	Intracellular water
BMR	Basal metabolic rate
RA	Right arm
LA	Left arm
TR	Trunk
RL	Right leg
LL	Left leg
