# Supplementary material for: Extracellular Water to Total Body Water Ratio in Septic Shock Patients Receiving Protocol-Driven Resuscitation Bundle Therapy
Source: J Clin Med. 2021 Jun 29;10(13):2917. doi: 10.3390/jcm10132917 (PMC8269068; doi:10.3390/jcm10132917)
Supplement: Supplementary file 1 [file jcm-10-02917-s001.zip › jcm-1234150-supplementary.pdf]

Supplementary S1. The protocol of bundle therapy for septic shock patients and compliance of our institution.

The protocol of bundle therapy for septic shock patients
• Initiation of crystalloid fluid (20-30cc/kg) rapid loading
• Obtain blood culture
• Administer intravenous broad-spectrum antibiotics
• Apply vasopressor if persistent hypotensive with systolic blood pressure <90 mmHg or mean arterial pressure <70 mmHg <u>during or after</u> fluid resuscitation to maintain MAP ≥ 65 mm Hg
• Measure lactate level and re-measure within 4hours

The compliance of the protocol of bundle therapy showed following diagram and tables.

Figure S1.1. The compliance of the protocol of bundle therapy according to each factor completion time

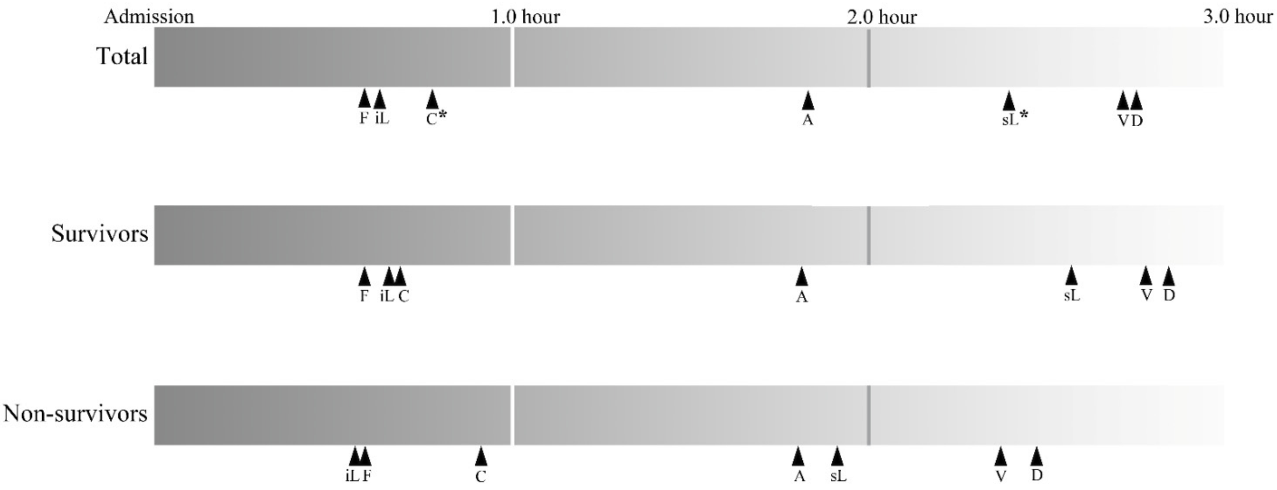

F, the timing of initiation of fluid loading; iL, the timing of obtained initial lactate; C, the timing of performing the first set of blood culture; A, the timing of administration of intravenous antibiotics; sL, the timing of obtained follow-up second lactate; V, the timing of applying vasopressors; D, the timing of completion of adequate fluid volume dripped.

Table S1.1. The compliance of the protocol of bundle therapy according to each factor completion time.

	Total	Survivors	Non-survivors	*p* value
F, Initiation of fluid (hours)	0.58 ± 0.60	0.58 ± 0.62	0.58 ± 0.57	0.993
iL, Initial lactate (hours)	0.62 ± 0.46	0.64 ± 0.46	0.56 ± 0.44	0.190
C, Blood culture (hours)	0.74 ± 0.60	0.68 ± 0.51	0.91 ± 0.78	0.022
A, IV antibiotics (hours)	1.81 ± 1.08	1.81 ± 1.07	1.80 ± 1.09	0.948
sL, Second lactate (hours)	2.40 ± 1.71	2.57 ± 1.87	1.92 ± 1.02	0.000

V, Vasopressor (hours)	2.69 ± 1.93	2.81 ± 1.95	2.37 ±1.84	0.104
D, Completion of fluid drip (hours)	2.75 ± 1.89	2.85 ± 2.09	2.47 ± 1.14	0.068

F, the timing of initiation of fluid loading; iL, the timing of obtained initial lactate; C, the timing of performing the first set of blood culture; A, the timing of administration of intravenous antibiotics; sL, the timing of obtained follow-up second lactate; V, the timing of applying vasopressors; D, the timing of completion of adequate fluid volume dripped. *$p<0.05$.

Considering that extreme values affect the mean value, the proportion of patients who performed bundle therapy within the expected time is as follows.

Table S1.2. The proportion of patients who performed bundle therapy within 3 hours.

	**Total**	**Survivors**	**Non-Survivors**	***p*-Value**
Initiation of fluid loading (%)	259 (99.2)	189 (99.0)	70 (100.0)	0.390
Lactate measurement (%)	261 (100.0)	191 (100.0)	70 (100.0)	NA
Blood culture (%)	257 (98.5)	189 (99.0)	68 (97.1)	0.292
Administration of IV antibiotics (%)	221 (84.7)	161 (84.3)	60 (85.7)	0.778
Apply vasopressors (%)	166 (63.6)	115 (60.2)	51 (72.9)	0.060

Table S1.3. The proportion of patients who performed bundle therapy within 1 hour.

	**Total**	**Survivors**	**Non-Survivors**	***p*-Value**
Initiation of fluid loading (%)	232 (88.9)	171 (89.5)	61 (87.1)	0.587
Lactate measurement (%)	226 (86.6)	165 (86.4)	61 (87.1)	0.874
Blood culture (%)	215 (82.4)	164 (85.9)	51 (72.9)	0.015
Administration of IV antibiotics (%)	62 (23.8)	44 (23.0)	18 (25.7)	0.652
Apply vasopressors (%)	55 (21.1)	37 (19.4)	18 (25.7)	0.266

Supplementary S2. Explanation and informed consent about study registration of patients with septic shock at the emergency department.

Version 1.2

Aim of this study registration

The advancement of medicine is made through researches through analysis of patients' data. The patient was diagnosed and treated for septic shock in the emergency department, Asan medical center, so we are going to seek consent to obtain the medical record information of the patient for the purpose of conducting a "study registration of patients with septic shock at the emergency department". If the patients agree to use the data, our researchers will make the following matters based on the this.

First, access the medical record information on diagnosis and treatment. Obtained information shall be kept confidential and will be used for research purposes only.

Second, it will be used for other research in the future. In other words, in the cases of other various clinical studies on septic shock are conducted in this hospital, our researchers will review the patient's medical record and conduct another research for medical advancement.

Eligibility for registration of this study

Eligibility for registration of this study is patients who have admitted emergency department and treated for septic shock at Asan medical center.

Possible risk from participating in the registration of this study

There is no risk that may arise from participating in the registration of our study. By participating in the registration of the study, the patient's personal information may be known to the outside, our researchers will do our best to protect the patient's personal information. Data will be stored by processing the personal identifier such as resident registration number, patient's identification number, into a substitute identifier, our researchers will strictly prevent being shown the personal identification in any case. In addition, we will try to prevent personal information from being leaked by using various protection ways. Data in research publications are expressed only for the entire study participant, and personal data are not displayed.

Possible benefit from participating in the registration of this study

There is no direct benefit that may arise from participating in the registration of our study. However, the medical information included in the registration of the study will be used to develop the treatment for septic shock patients and will be good for septic shock patients in the future.

Payment from the patients who were participated in the registration of this study

There is no additional payment of the cost to the patient by participating in the registration of our study.

Compensation to the patients who were participated in the registration of this study

There is no compensation to the patients who were participated in the registration of this study.

Information on medical records included in the registration of this study

The information on medical records included in the registration of this study is the patient's past, present, and future information related to septic shock. Since medical conditions and treatments not related to septic shock can also affect septic shock treatment, medical information from the past, present, and future will be included in the registration of study.

Persons with access to personal identifiable information and duration of storage of medical records

If you agree to participate in the clinical study, various medical information such as your age, gender, medical history, surgical experience, medication history, etc., and research-related test results are recorded and stored in a specific format (paper or computer). This information will be shared by researchers participating in the study and it is managed through the use of a lock or password so that no other access except researchers who were permitted. The collected data will be crushed and discarded three years after the study is completed. However, the clinical research review committee of our institute and the subject protection center may review the information to confirm the correct progress of the study. Even if you stop participating in the study while progressing the study, your information collected until the point of the stop will be used for the study. In addition, all information obtained in this study will be processed in accordance with national and local laws. By signing this agreement, you consent to all of the above.

Voluntary participation

Participation in the registration of the septic shock study described above is voluntary. Our researchers will always do our best to treat patients regardless of participation.

Suspension of participation in registration

While participating in the study, you would like to learn more about this study or if you have any questions, you can contact the research coordinator.

A research manager: Won Young Kim, contact: 82-2-3010-3350

If you have any questions about your welfare and rights as a participant in the clinical study while participating in the clinical study; or if you would like to consult with someone who is not directly related to the research, please contact the number below.

Subject protect center: 82-2-3010-7161

Clinical research review committee of Asan medical center: 82-2-3010-7166

I have listened to and understood all explanations related to this clinical study, and I have heard enough answers to all inquiries. After taking enough time to think, I would like to participate in the above study by voluntary intention. In addition, I permit to use and share my health information, I know I will receive a copy of the consent form.

Name of study subject ______________ / Signature _____________ /

Date of consent __________________

Name of the representative of study subject ______________ /

Relationship with the subject _______________ / Signature ____________ /

Date of consent ___________________

I, as the clinical researcher, confirm that the research subject or the research subject's representative were provided sufficient explanation by the clinical researcher.

Name of clinical researcher ______________ / Signature _____________ /

Date of consent __________________

Supplementary S3. BIA device and protocol.

The principle of muti-frequency BIA method

Water is considered the only electrically conductive component in the body. BIA device introduces an electrical current throughout the body, then the impedance of water can be measured when the current passes through the body. With this impedance value, the volume of body water is calculated based on Ohm’s Law (Resistance (Ω)=Voltage (V)/Intensity (A)).[20]

The ability of the current to pass through cell membranes varies according to the frequency of the current. Low-frequencies current relies on the conductive properties of extracellular water (ECW) while high-frequencies current relies on both intracellular water (ICW) and ECW conductive properties.[20] The difference in frequencies of current is related to their abilities to penetrate cell membranes. Low-frequency current cannot penetrate the cell wall and remain in the extracellular fluid, high-frequencies current can pass through the cell membrane. Thus, ECW can be measure at low frequencies, total body water (ECW + ICW) can be estimated at high frequencies.

Also, fat-free mass (FFM) is composed of a high electrolyte and water content, which produces high conductivity with low impedance, while fat mass (FM) contains low electrolyte and water content, creating a higher impedance resulting in low conductivity. The difference between resistance and impedance values, which is negligible at low frequencies but increases at higher frequencies. [21]

In the late 1980s, the BIA method was a single-frequency only at only 50kHz that yielded inaccurate results and required the statistical correction of measurements. Through understanding the cause of BIA’s inaccuracy and developing the technology, a new developed BIA device with multi-frequency measurement for more accurate analysis of intracellular water and extracellular body water by using multiple broadband frequencies (1 to 1,000 kHz).

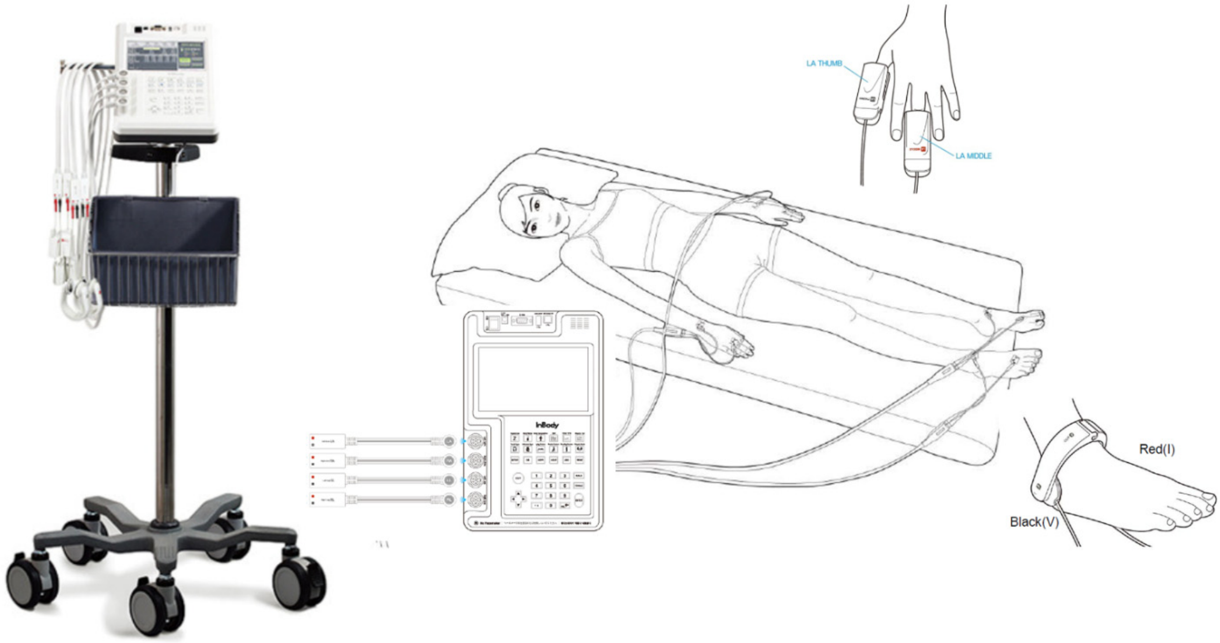

BIA device (inbody S10) and electrodes.

The way to connect the cables of electrodes to the device and the way to place electrodes on examinee were depicted in the illustration. (The English version of InBody S10 user's manual ⓒ1996- InBody Co.)[19]

Body composition analyses by the inbody S10 device were carried out in the following order.

- Patients recognized as septic shock are required to maintain the supine posture at least 10 minutes before applying the device. If the clothing patients or sheet are wet by sweat or urine, remove them and exchange to dry sheet or patient's clothes.
- Examiner brings the device and places it on the firm and flat floor. When the examiner takes a look around the bed if there are some devices or tools that generate an electrical signal or heat, move them away from the BIA device.
- Connect the adapter cable to the power input port and turn on the switch of the device.
- Select electrode type, examinee's posture type, and then insert personal information such as patients ID, weight, height, age, and sex on the LCD screen.
- Four units of touch-type hand electrodes with 2 units per hand are placed on the thumb and middle fingers of both hands, respectively. Place two units of touch-type foot electrodes between ankle bone and heel. Make the examinee keep both arms abducted naturally to a 15-degree angle away from the trunk with a supine position.
- Press the 'Enter' button to start the test. The examiner should make sure that the examinee maintains his/her position during the test. Values in body composition including the ECW/TBW ratio are calculated and saved automatically. After the test is completed, a "Test Complete" message will appear on the screen.
- Press the 'Exit' button to close the results screen.

Utilizing the BIA device is easy and simple, so the examiner does not require a high degree of technical skill to operate. The average time for preparation of device, adjustment, and measurement is enough about 10 minutes. Body composition analyses were performed during resuscitation for septic shock. All BIA tests were carried out by interns who were familiar with how to use the device by the manufacturer's protocol.

References

19. InBody S10 User's Manual. Available online: InBodyS10_CDmanual_Eng_E.pdf (accessed on 26 June 2021).

20. Gibson, A.L.; Holmes, J.C.; Desautels, R.L.; Edmonds, L.B.; Nuudi, L. Ability of new octapolar bioimpedance spectroscopy analyzers to predict 4-component–model percentage body fat in Hispanic, black, and white adults. *Am. J. Clin. Nutr.* 2008, *87*, 332–338.

21. Leahy, S.; O'Neill, C.; Sohun, R.; Jakeman, P. A comparison of dual energy X-ray absorptiometry and bioelectrical impedance analysis to measure total and segmental body composition in healthy young adults. *Eur. J. Appl. Physiol.* 2012, *112*, 589–595.
